# Shexiang Tongxin dropping pill protects against isoproterenol-induced myocardial ischemia *in vivo* and *in vitro*

**DOI:** 10.18632/oncotarget.22440

**Published:** 2017-11-14

**Authors:** Jianyong Qi, Wenjun Pan, Yafang Tan, Jiaru Luo, Dancai Fan, Juan Yu, Jiashin Wu, Minzhou Zhang

**Affiliations:** ^1^ AMI Key Laboratory of Chinese Medicine in Guangzhou, The Second Affiliated Hospital of Guangzhou University of Chinese Medicine, Guangdong Province Hospital of Chinese Medicine, Guangzhou 510006, China; ^2^ Intensive Care Research Team of Traditional Chinese Medicine, The Second Affiliated Hospital of Guangzhou University of Chinese Medicine, Guangdong Province Hospital of Chinese Medicine, Guangzhou 510006, China; ^3^ Animal Center, The Second Affiliated Hospital of Guangzhou University of Chinese Medicine, Guangdong Province Hospital of Chinese Medicine, Guangzhou 510006, China; ^4^ Department of Pharmaceutical Sciences, College of Pharmacy, Northeast Ohio Medical University, Rootstown, Ohio 44272, USA

**Keywords:** myocardial ischemia, isoproterenol, signaling pathway, rat, Shexiang Tongxin dropping pill

## Abstract

Shexiang Tongxin dropping pill (STDP) is a formulae of Chinese Medicine commonly used to treating angina pectoris in China. However, its mechanism of action is still yet unclear. This study investigated the roles of STDP on myocardial ischemia injury. We constructed a rat model of myocardial injury (isoproterenol subcutaneous injection, i.h, 85 mg/kg/day for 2 days), and compared among 4 groups: CON (control), ISO (ischemic injury model), MET (metoprolol), and STDP. Serum contents of Troponin I (cTnI), creatine kinase (CK), CK-MB, lactate dehydrogenase (LDH), alpha-hydroxybutyric dehydrogenase (α-HBD), and Aspartate Aminotransferase were detected and five STDP doses (1, 10, 100, 1000 and 10000 mg/kg/day) were chosen to obtain a dose-response curve. Western-blot was used to detect phosphorylations of extracellular signal-regulated kinase 1/2 (ERK1/2), protein kinase B (AKT), and camodulin kinase II (CamkII). Furthermore, an ERK1/2 inhibitor PD98059, a phosphatidylinositol-3-kinase inhibitor, LY294002, and a CamKII inhibitor, KN-93 were administered i.h. Results: cTnI, CK, CK-MB, α-HBD, and LDH were significantly lower in STDP than ISO (*P*<0.05). STDP exhibited a dose-dependent effect with a half maximal inhibitory concentration of 42 mg/kg/day. Phosphorylation of ERK1/2 was enhanced in the STDP group (vs. ISO, *P*<0.05), while AKT and CamkII were not changed. Further, the protective effects of STDP were offset by PD98059 administration i.h. In conclusion, STDP protected against the ISO-induced myocardial ischemic injury via an ERK1/2 signaling pathway, which provided a mechanism to support clinical applications of STDP as treatment for ischemic heart disease.

## INTRODUCTION

Myocardial ischemia and infarction are the leading causes of morbidity and mortality in U.S. [1] and are also becoming major causes of death worldwide due to prevalence of obesity, high blood pressure, and unhealthy lifestyles [2]. Although percutaneous coronary intervention (PCI) is effective in reducing the consequences of myocardial ischemia and infarction, PCI procedures also frequently cause reperfusion injury. Moreover, sudden cardiac death can occur before patients have the chance of receiving PCI operation. Therefore, drug treatment is still a convenient and important method to rescue patients from acute myocardial ischemia and infarction. Despite advances in basic research and clinical improvements, current drug therapies are still insufficient to manage and treat the disease.

ShexiangTongxin dropping pill (STDP), an herb formula of Traditional Chinese Medicine, has been widely used for treating angina pectoris in Chinese clinics and hospitals during the last 2 decades. It was reported that STDP could effectively alleviate unstable angina pectoris and reduce major adverse cardiovascular events in patients when combined with conventional therapies [3]. It was also demonstrated that STDP could enhance cardiac function in patients with heart failure [4].

**Figure 1:**
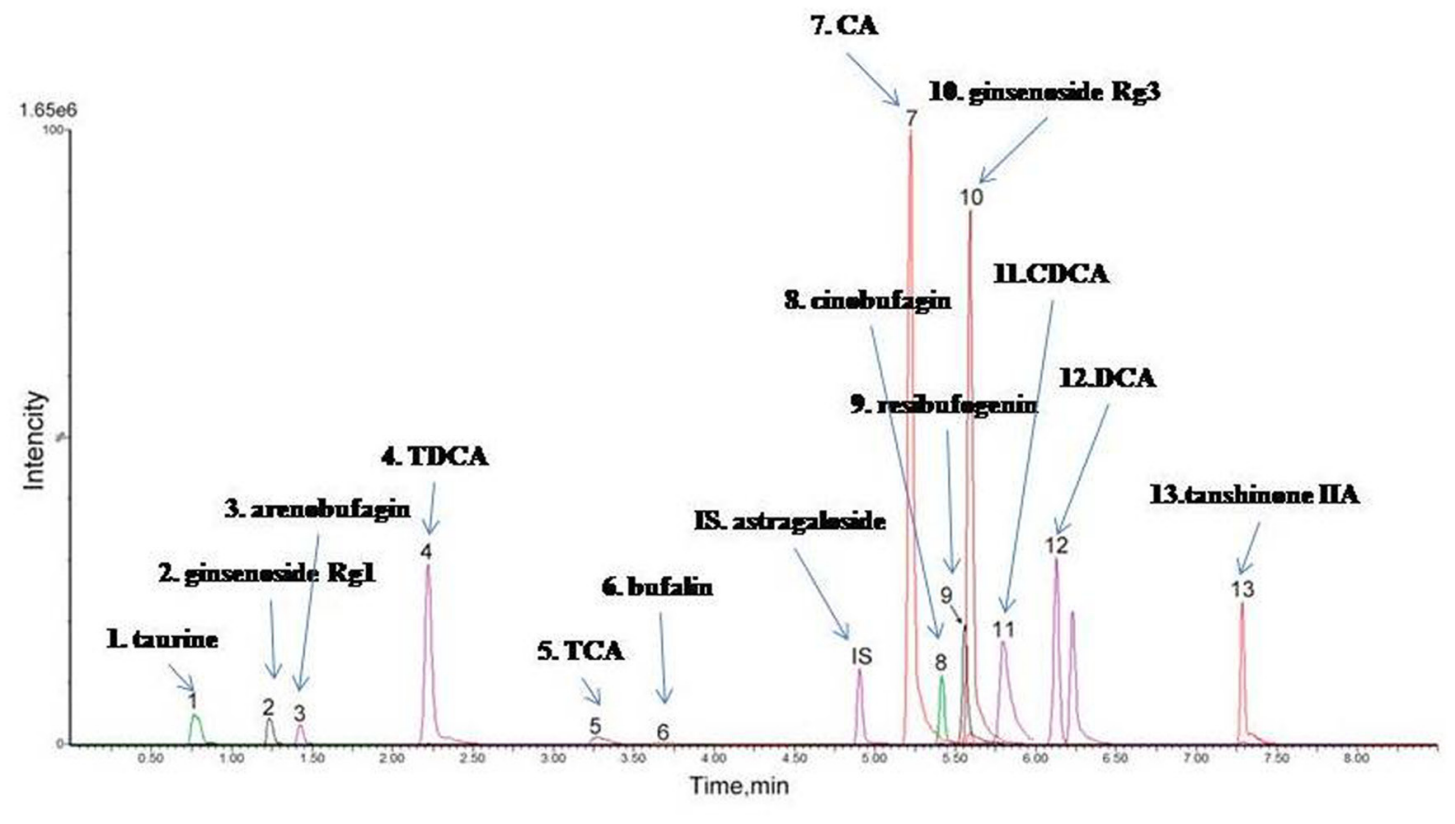
Thirteen major chemical constituents in STDP MRM chromatograms of 13 markers and internal standard: (1) taurine, (2) ginsenoside Rg1, (3) arenobufagin, (4) TDCA, (5) TCA, (6) bufalin, (7) CA, (8) cinobufagin,(9) resibufogenin, (10) ginsenoside Rg3, (11) CDCA, (12) DCA, (13) tanshinone IIA,(IS) astragaloside.

Although STDP is a complex herb formula, ultra-performance liquid chromatography coupled with triple-quadrupole tandem mass spectrometry (UPLC-QqQ-MS/MS) Quantitative Analysis revealed 13 major chemical constituents in STDP: ginsenoside Rg1, ginsenoside Rg3, cinobufagin, arenobufagin, bufalin, resibufogenin, tanshinone IIA, taurine, astragaloside, tauroursodeoxycholic acid, taurocholic acid, cholic acid, deoxycholic acid, and chenodeoxycholic acid. (Figure 1) [5]. Artificial musk administration was reported to protect against myocardial ischemic injury in animal studies [6]. Fel Ursi showed various pharmacological activities, such as antimicrobial, anti-inflammatory, treating hypertension, liver fibrosis, dyslipidemia, thrombosis, and tumor growth, etc [7, 8].

Despite broad applications of STDP in treating angina pectoris in Chinese clinics and hospitals, seldom is reported about the underlying mechanisms of STDP treating myocardial ischemic injury. The present study investigated mechanisms of STDP's cardio-protective effects using an ISO-induced myocardial ischemic injury model in rats.

## RESULTS

### STDP significantly reduced the ISO-induced myocardial ischemia *in vivo*

**Figure 2:**
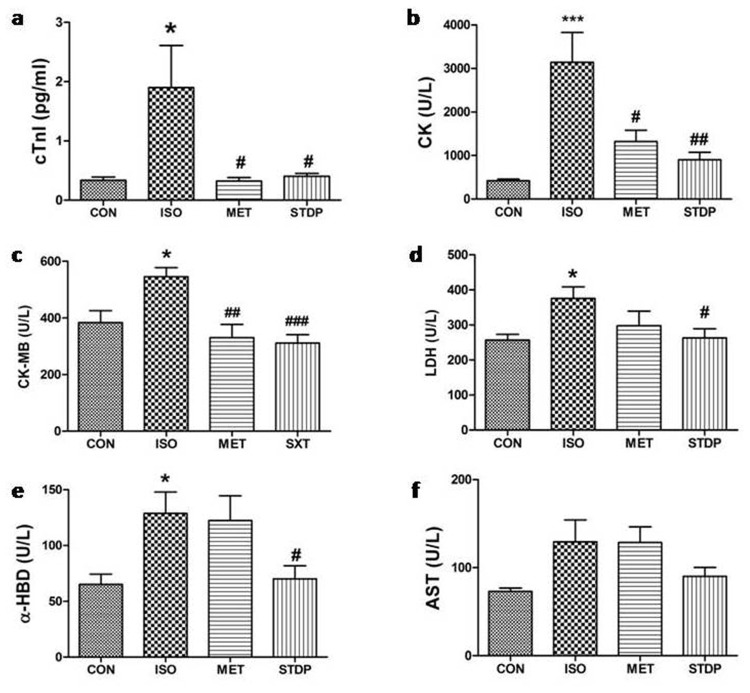
Effects of STDP on serum indices of myocardial injury in rats Plasma levels of **(a)** cTnI, **(b)** CK, **(c)** CK-MB, **(d)** LDH, **(e)** α-HBD, **(f)** AST among the4 study groups (CON, ISO, MET, and STDP) were measured by ELISA methods. ^*^ P < 0.05, ^***^ P < 0.001, compared with CON; ^#^ P < 0.05, ^##^ P < 0.01, ^###^ P < 0.001, compared with the ISO group.

To explore the effects of STDP on myocardial injury *in vivo*, we created a rat ischemia model by ISO subcutaneous injection (i.h, 85 mg/kg/day for 2 consecutive days). As shown in Figure 2 and Table 1, serum content of cTnI and kinase activities of CK, CK-MB, LDH, and α-HBD were increased in the ISO group (positive myocardial injury model), and decreased in MET group (positive drug treatment) (vs. control, *P*<0.05), which demonstrated that the ISO-induced myocardial injuries were stable and replicable. Moreover, all indexes of myocardial injury (cTnI, CK, CK-MB, LDH, and α-HBD) in the STDP group were decreased (vs. ISO, *P* < 0.05), which showed that STDP alleviated the ISO-induced myocardial injury *in vivo*.

**Table 1:** Elisa data of four groups

	CON	ISO	MET	STDP
**cTnI(pg/ml)**	**0.335±0.06**	1.900±0.71^*^	**0.322±0.06**^#^	**0.403±0.05**^#^
**CK(u/l)**	**422.4±36.0**	3140.±686^***^	**1322.±261**^#^	**900.4±175.1**^##^
**CK-MB(u/l)**	**383.8±41.9**	545.9±31.7^*^	**330.6±46.7**^##^	**311.4±29.40**^###^
**LDH(u/l)**	**256.8±16.9**	375.7±32.9^*^	**297.5±42.1**	**262.5±26.50**^#^
**α-HBD(u/l)**	**65.14±9.20**	128.9±19.1^*^	**122.3±22.3**	**70.13±11.63**^#^
**AST(u/l)**	**73.00±3.99**	**129.4±24.9**	**128.5±17.8**	**90.00±10.26**

Note: ^*^P < 0.05, ^***^P < 0.001, compared with the control group (CON). ^#^*P* < 0.05, ^##^*P* < 0.01, ^###^*P* < 0.001, compared with the ISO group.

**Figure 3:**
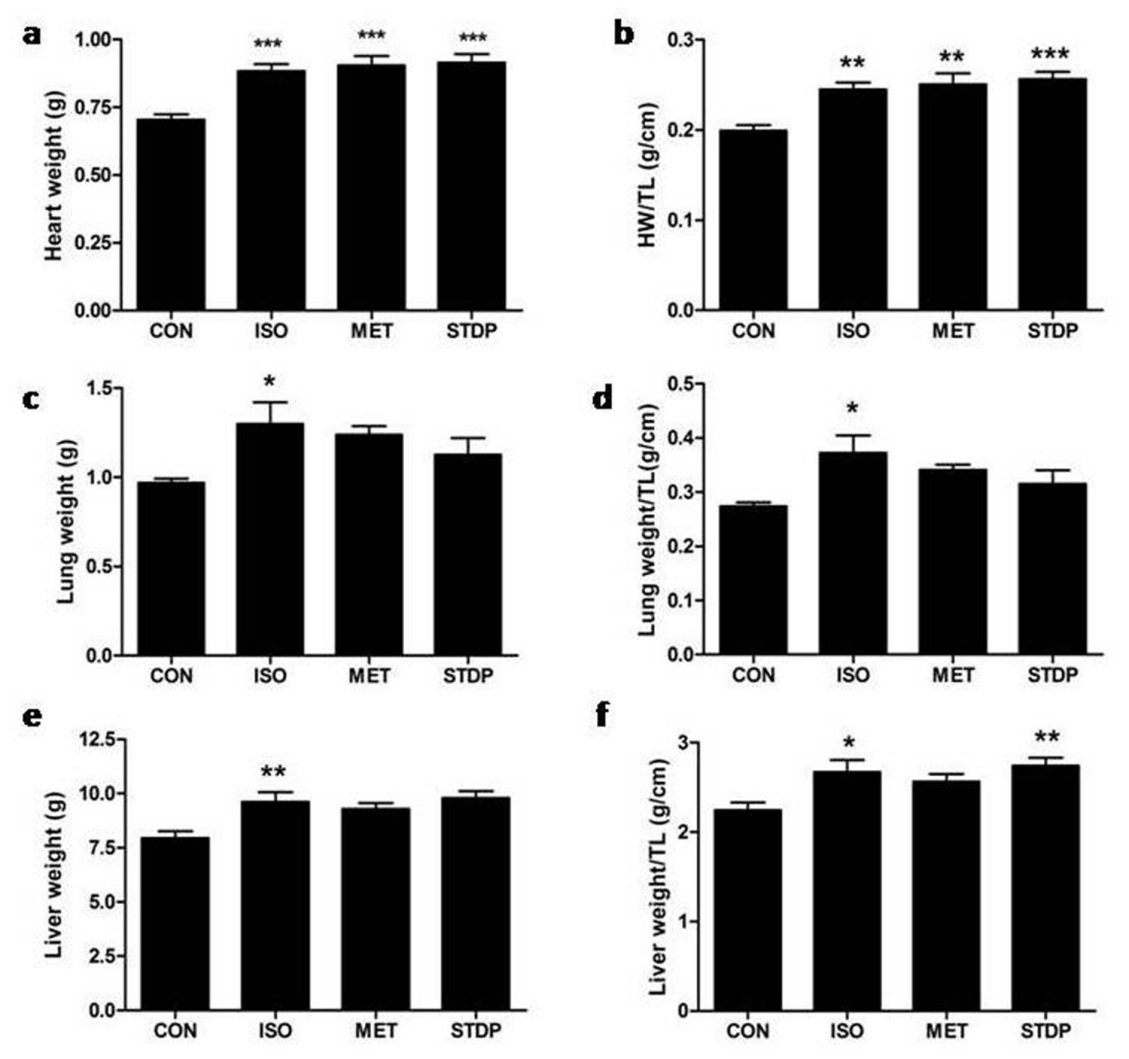
Anatomic data among the four groups **(a)** Heart weight (HW), **(b)** Ratio of HW/TL, **(c)** Lung weight, **(d)** Ratio of Lung weight/TL, **(e)** Liver weight, **(f)** Ratio of Liver weight/TL were measured. Data are presented as mean ± S.E.M. ^*^ P < 0.05, ^**^ P < 0.01, ^***^ P < 0.001 compared with the CON group.

Subsequently, rats were sacrificed and their heart, lung, and liver were harvested and weighted (Figure 3, Table 2, and Supplementary Figures 1-2). Compared with the CON group, there were significantly increases in heart weight (HW), ratio of heart weight/body weight (HW/BW), and ratio of heart weight/tibial length (HW/TL) in the ISO group (*P* < 0.001), which confirmed that cardiac hypertrophy was formed after ISO treatment. Lung weight, ratio of Lung W/BW, and Lung W/TL were increased (vs. control, *P* < 0.05), suggesting that lung edema was formed after ISO stimulation. Liver weight, ratio of Liver W/BW, and Liver W/TL were increased (vs. control, *P* < 0.05), suggesting that right heart failure was formed in the ISO group. Thus, pathological cardiac remodeling was formed after ISO stimulation. Compared with the ISO group, STDP and MET group did not have significant differences in cardiac hypertrophy. However, BNP were significantly reduced in the STDP group than ISO group (*P*<0.05, Supplementary Figure 3), which suggested that STDP partly alleviated acute pathological cardiac remodeling.

**Table 2:** Anatomical data of four groups

	CON	ISO	MET	STDP
**BW(g)**	**217.9±4.35**	**210.9±4.73**	**210.8±5.74**	**211.6±1.82**
**Heart(g)**	**0.705±0.02**	0.884±0.03^***^	0.904±0.04^***^	0.916±0.03^***^
**Lung(g)**	**0.969±0.02**	1.299±0.12^*^	**1.238±0.05**	**1.127±0.09**
**Liver(g)**	**7.946±0.31**	9.621±0.45^**^	**9.291±0.27**	9.786±0.32^**^
**TL(cm)**	**3.542±0.03**	**3.609±0.03**	**3.624±0.05**	**3.572±0.02**
**HW/BW**	**3.234±0.06**	4.201±0.15^***^	4.319±0.26^***^	4.323±0.12^***^
**Lung/BW**	**4.456±0.12**	6.220±0.66^*^	**5.868±0.14**	**5.325±0.43**
**Liver/BW**	**36.51±1.30**	45.53±1.54^**^	44.31±2.13^*^	46.29±1.57^***^
**HW/TL(g/cm)**	**0.199±0.01**	0.245±0.01^**^	0.250±0.01^**^	0.256±0.01^***^
**Lung/TL(g/cm)**	**0.274±0.01**	0.373±0.03^*^	**0.341±0.01**	**0.315±0.03**
**Liver/TL(g/cm)**	**2.240±0.09**	2.670±0.13^*^	**2.567±0.08**	2.741±0.09^**^

Note: ^*^P < 0.05, ^**^P < 0.01, ^***^P < 0.001, compared with the control group (CON).

### STDP alleviated myocardial ischemic injury dose-dependently in rats

**Figure 4:**
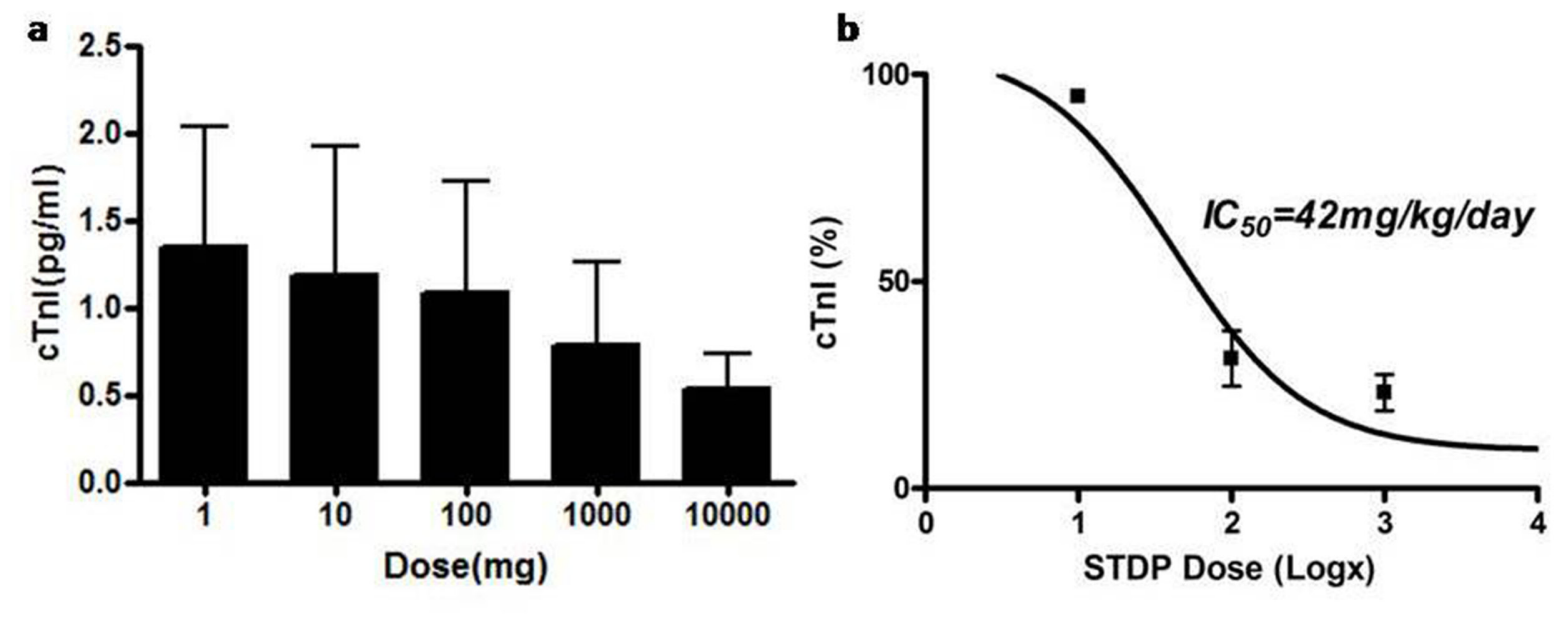
STDP inhibited serum cTnI expression dose-dependently in rats **(a)** cTnI were detected in different STDP concentrations from 1 to 10000 mg/kg/day by ELISA analysis. **(b)** Dose-dependent curve was obtained to normalize the inhibitive effect of STDP on rats' myocardial ischemic injury, IC50 = 42 mg/kg/day.

To determine dose-response relationship of the effects of STDP on ISO-induced myocardial ischemic injury in rat, an ELISA method was implemented and cTnI was chosen as the marker of myocardial injury due to its high accuracy, super sensitivity and high specificity. Five doses of STDP (1, 10, 100, 1000, and 10000 mg·kg^-1^·day^-1^) were selected. As shown in Figure 4a. myocardial injury was not reduced by 1 or 10 mg/kg/day STDP, while significantly inhibited by 100 mg/kg/day STDP. Over 100 mg/kg/day, STDP protected against ISO-induced myocardial injury with statistical significance (*P* < 0.05). The dose-response curve followed a sigmoidal shape (Figure 4b) with a half-maximal response at 42 mg·kg^-1^·day^-1^. Therefore, STDP protected against the ISO-induced myocardial injury dose-dependently in rats.

### Phosphorylation of ERK1/2 was increased in ischemic cardiac tissue *in vivo* and *in vitro*

**Figure 5:**
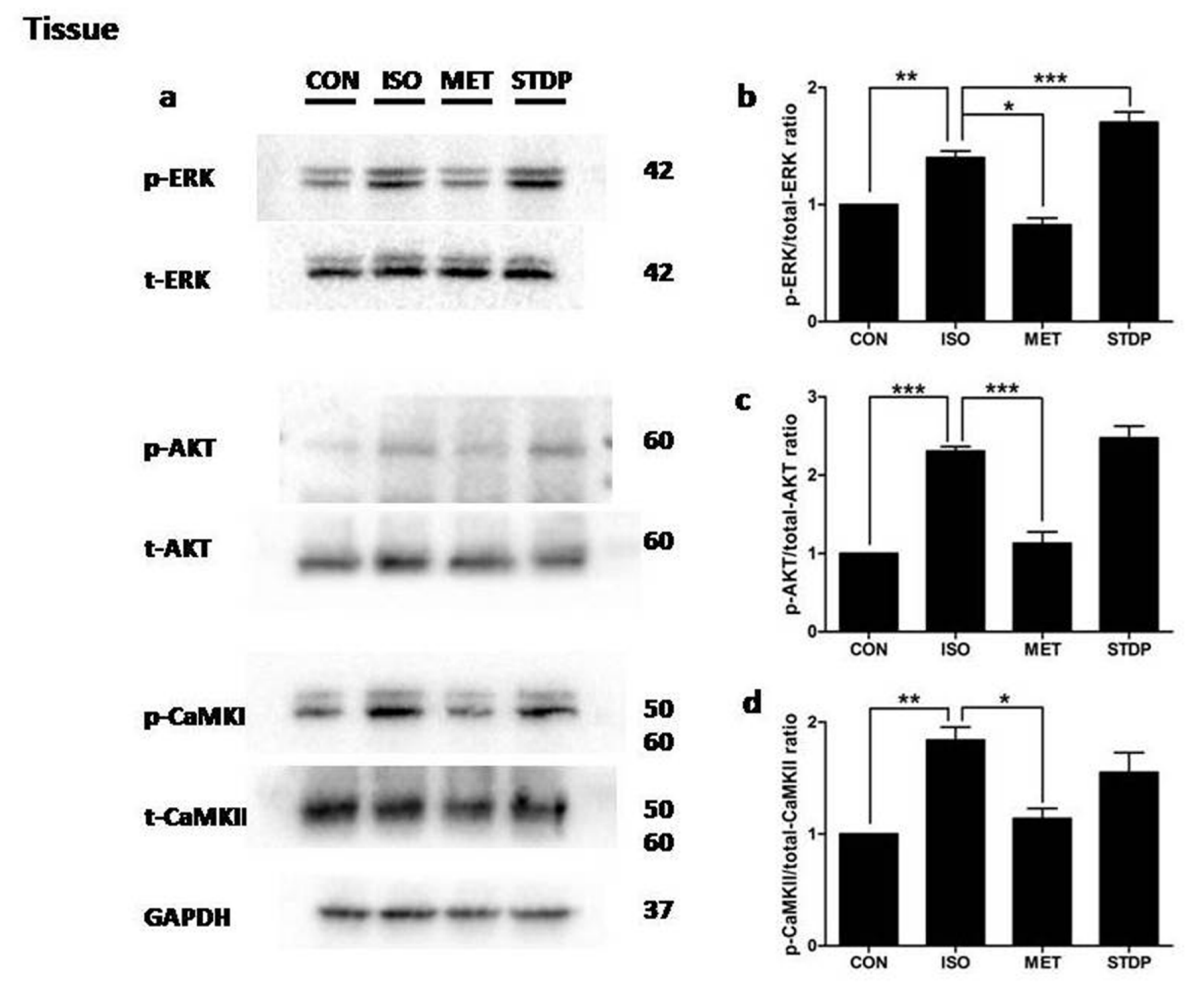
STDP enhanced ERK1/2 phosphorylation after ISO stimulation *in vivo* **(a)** Western blotting was performed to detect phosphor-ERK1/2, AKT, and CaMKII. Total proteins and GAPDH were used as loading controls. **(b)** p-ERK1/2, **(c)** p-AKT, **(d)** p-CaMKII (mean ± SEM, n = 3) were expressed as fold changes from phosphorylated and total proteins. ^*^P < 0.05, ^**^P < 0.01, ^***^P < 0.01.

To investigate potential mechanisms of STDP protection on myocardial ischemic injury, we studied three major signaling pathways in myocardial ischemic injury, ERK1/2, AKT, and CaMKII [9–12]. Some researchers reported that the phosphorylation of ERK1/2 was increased in ischemic myocardium and played a harmful effect, while others reported that ERK1/2 played a protective role in the RISK pathway [13–16]. Therefore, it is hard to differentiate that the phosphorylation of ERK1/2 was beneficial and/or harmful to ischemic myocardium. In the present study, Western blot analysis showed that phophsrylations of ERK1/2, AKT, and CaMKII were all enhanced after ISO stimulation (vs. CON, *P*<0.05, Figure 5). Furthermore, phosphorylation of ERK1/2, AKT, and CaMKII were inhibited after metoprolol precubation (MET vs ISO group, P<0.05) in consistence with previous publications and suggested stability and reproducibility of our ISO-induced myocardial injury model. Interestingly, phosphorylations of ERK1/2 were significantly increased in the STDP group, (vs. ISO group, *P* < 0.05), whereas both AKT and CaMKII phosphorylations were not significantly changed (vs. ISO group, P>0.05). Together, these data demonstrated activation of ERK1/2 signaling pathway, but not AKT and CaMKII, by STDP stimulation in its protection against myocardial ischemic injury.

**Figure 6:**
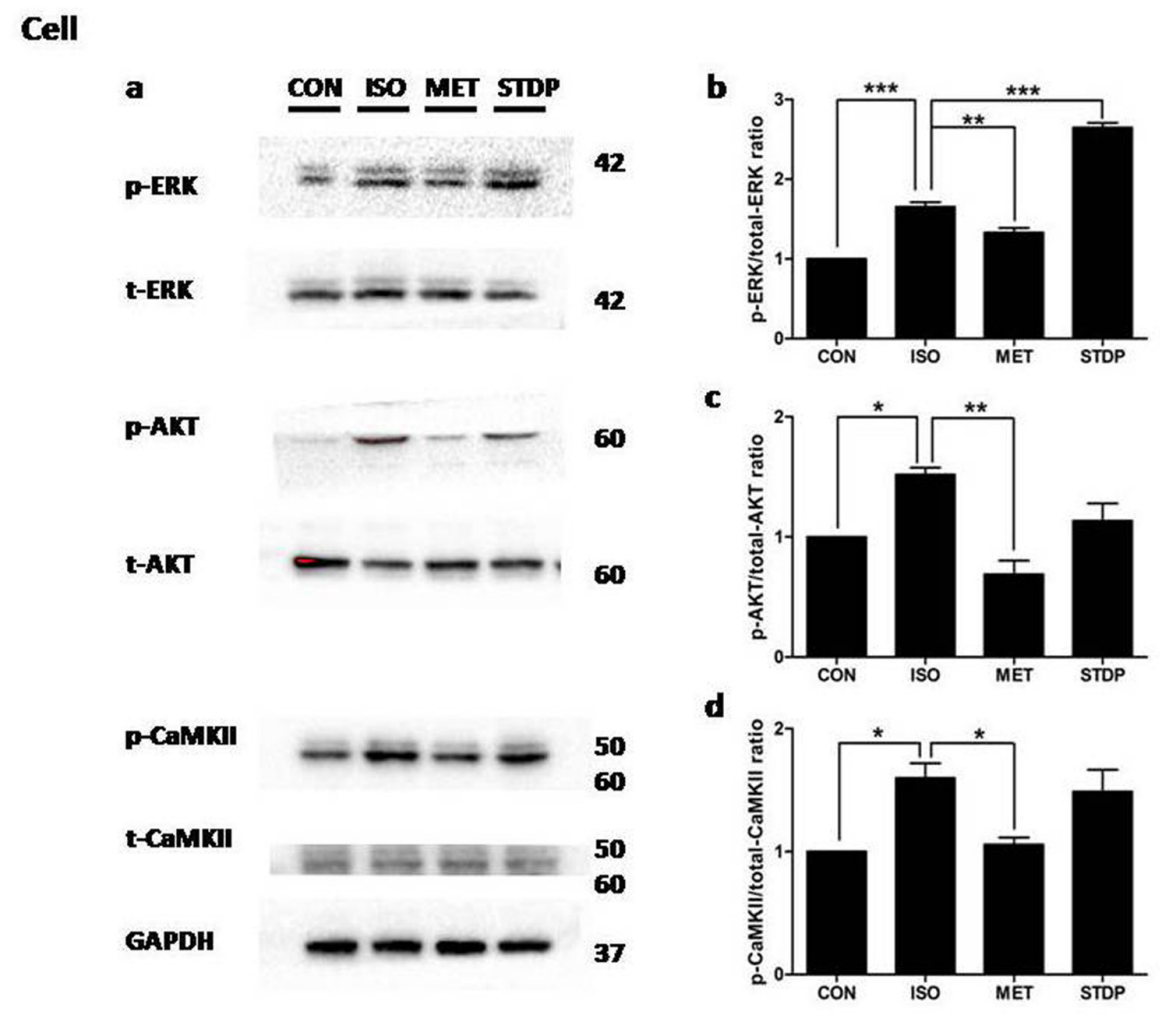
STDP enhanced ERK1/2 phosphorylation *in vitro* **(a)** Western blotting was performed to detect phosphor-ERK1/2, AKT, and CaMKII. Total proteins and GAPDH were used as loading controls. **(b)** p-ERK1/2, **(c)** p-AKT, **(d)** p-CaMKII (mean ± SEM, n = 3) were expressed as fold changes from phosphorylated and total proteins. ^*^P < 0.05, ^**^P < 0.01, ^***^P < 0.01.

To further validate the *in vivo* experiments, *in vitro* rat H9C2 cardiomyocytes were cultured to simulate ischemia injury by ISO pre-incubation (1μM ISO for 1.5 hours, Figure 6) followed by metoprolol or STDP stimulation. Western blot analysis showed enhancement of ERK1/2 phosphorylation after STDP stimulation (vs. ISO group, *P*<0.05, Figure 6a-6b). The phosphorylations of AKT and CaMKII were not changed significantly *(vs. ISO group, P >* 0.05, Figure 6c-6d), consistent with the *in vivo* data in rat cardiac tissue. Together, both *in vivo* and *in vitro* data supported that STDP activated ERK1/2 phosphorylation so as to protect against ISO-induced myocardial ischemic injury.

### PD98059, a ERK1/2 inhibitor, attenuated the protective effects of STDP in ischemic ischemia tissue in rats

**Figure 7:**
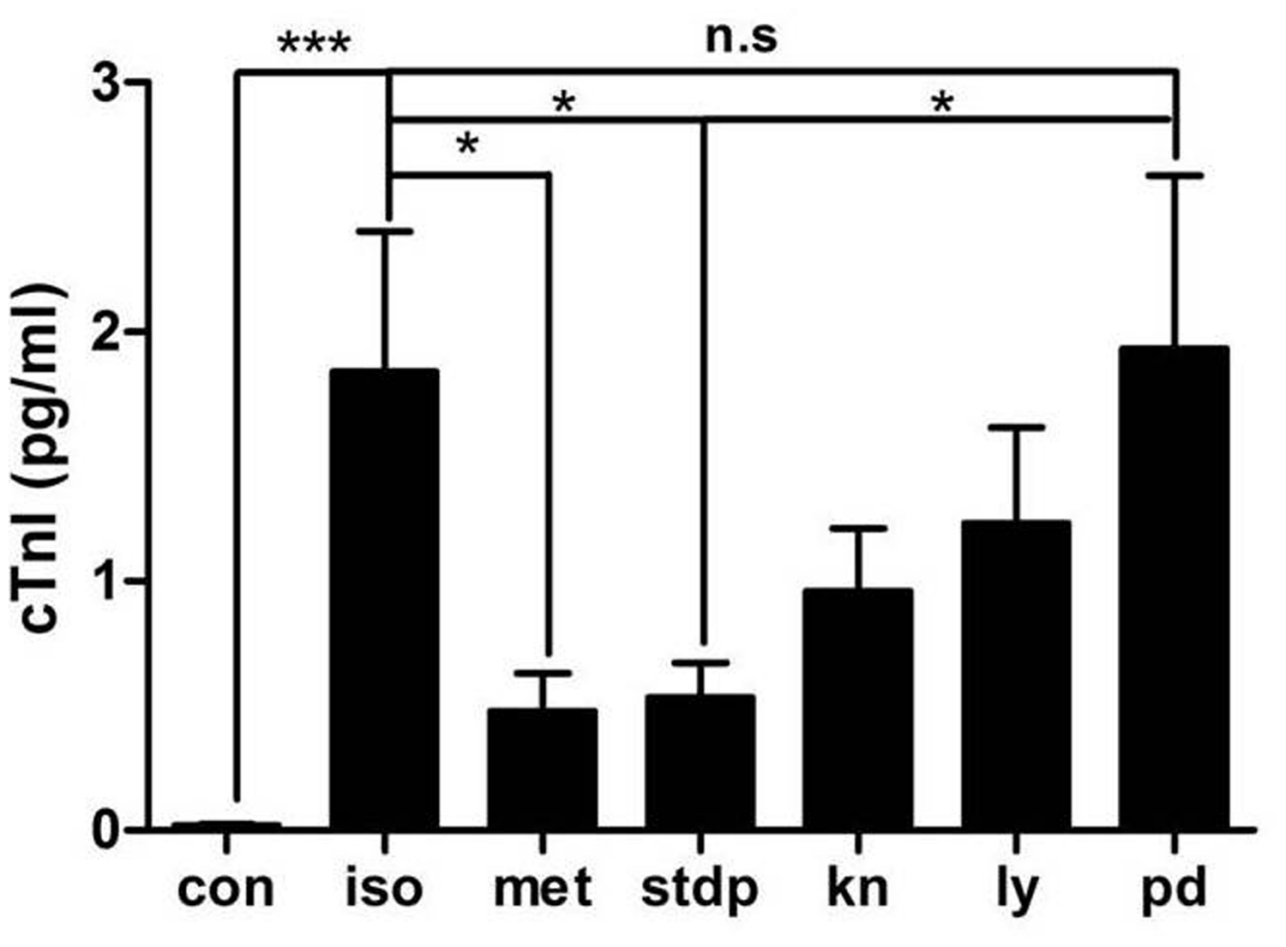
The protective effect of STDP on ISO-induced myocardial ischemic injury was diminished after PD98059 stimulation *in vivo* Plasma levels of cTnI among the 7 study groups (CON, ISO, MET, STDP, KN, LY, and PD) were measured by ELISA methods. ^*^ P < 0.05, ^**^ P < 0.01, ^***^ P < 0.001, n.s.: no significant difference.

To confirm the role of ERK1/2 phosphorylation in STDP's protective effects against the ISO-induced myocardial ischemic injury, we administrated PD98059 (a ERK1/2 inhibitor, PD), LY294002 (a PI3-K/AKT inhibitor, LY), and KN-93 (a CaMKII inhibitor, KN) i.h, after ISO and STDP stimulation. As shown in Figure 7, cTnI was increased in the ISO group and decreased in MET and STDP groups, in consistence with our previous results. However, cTnI in the PD group were significantly increased compared with STDP group (*P* < 0.05), and no significantly change compared with ISO group (*P* > 0.05), which suggested that the STDP’s protection diminished after inhibiting the activation of ERK1/2 (PD group) whereas STDP protective effects was still exist even after KN and LY intervention (*P*<0.05). Thus, both pharmacological and molecular methods supported the conclusion that STDP protected against myocardial injury via an ERK1/2-dependent signaling pathway.

**Figure 8:**
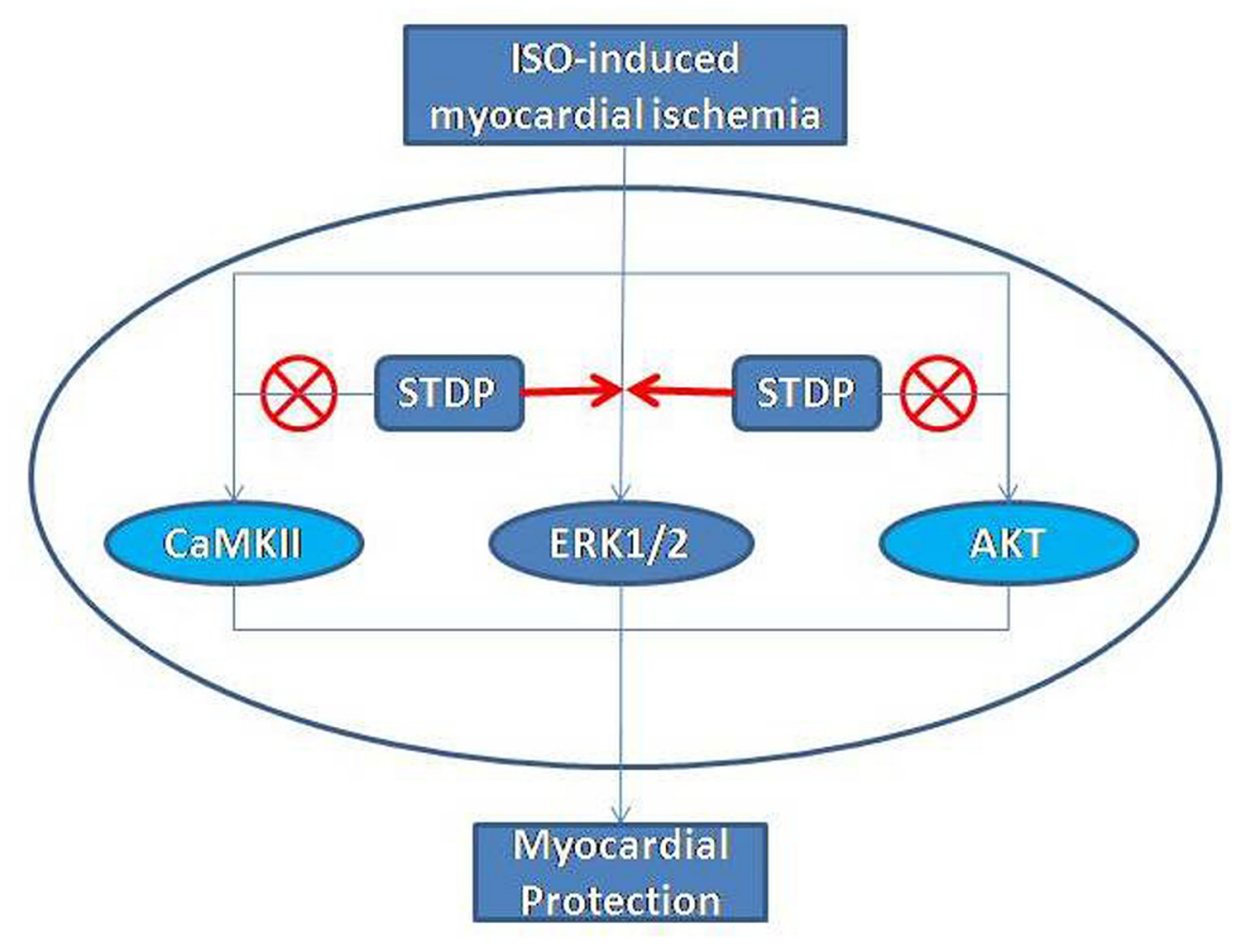
A model of pathways in the cardio-protection of STDP against ISO-induced myocardial ischemic injury Isoproterenol induced myocardial ischemic injury in rats. STDP enhanced the phosphorylation of ERK1/2, but neither AKT nor CaMKII signaling pathway, leading to decrease myocardial injury. ⊗ Denotes non-participation of the signaling pathways.

Based on the above observations, we formulated following work model (Figure 8): The ISO-induced myocardial ischemia was associated with enhanced phosphorylations of ERK1/2, AKT, and CaMKII. STDP further activated the ERK1/2 phosphorylation, but neither AKT nor CaMKII, to protect against ISO-induced myocardial ischemic injury.

## DISCUSSION

This is the first study to investigate the dose-dependent protective effects of STDP against myocardial ischemia injury in rat. We demonstrated that ERK1/2 inhibition by PD98059 abolished the STDP-induced cardio-protection, suggesting that STDP’s protection against myocardial ischemic injury was via an ERK1/2-dependent signaling pathway. These studies provided direct experimental support to STDP application in clinics and hospitals.

We constructed myocardial ischemic injury model by ISO i.h administration in rats. High dose ISO induced pathological changes, such as hypoxia, necrosis, and inhibition of cardiac function, similar to the changes in the AMI region of human heart [17]. As shown in Figure 2, cTnI, CK, CK-MB, LDH, and α-HBD were increased in the ISO-administrated rats. Patel et al. [18] reported that the ISO-induced cardiac pathological remodeling in rats might be due to the increased water content, edematous intramuscular space and extensive necrosis of inflammatory cardiac muscle. STDP inhibited the ISO-induced myocardial injury and reversed pathological cardiac remodeling, which further supported the role of STDP in protecting heart against myocardial ischemic injury.

Mechanisms of myocardial ischemia injury are complex. One of the most classical protective mechanisms of ischemic injury is RISK signaling pathway. ERK1/2 and AKT proteins were shown to play key roles in the RISK pathway [15]. It was reported that the phosphorylation of ERK1/2 was increased in ischemic myocardium, leading to cardiomyocytes apoptosis, and thus, played a harmful effect in the process of myocardial ischemia [13, 14]. More publications supported that ERK1/2 protein played a key role in the RISK pathway and was one of the most classical protective mechanisms of ischemic injury [15, 16]. In addition, it was reported that bidirectional control of ERK1/2 function in the pituitary provided a key mechanism for endocrine gene control [19]. Therefore, it is not as simple to label either a beneficial or harmful role of the phosphorylation of ERK1/2 to ischemic myocardium. In the present study, our data indicated that the phosphorylation of ERK1/2 was harmful in a lower expression while played a protective role in a relative higher expression, thus, bidirectional controls of ERK1/2 function in the ischemic myocardium in rats.

Recently, CaMKII was studied as a vital molecule in pathological cardiac remodeling [20, 21]. Considering pharmacological inhibition of CaMKII and activation of the ERK1/2 and PI3K/AKT protected cardiomyocytes from apoptosis during simulated ischemia *in vitro*, we chose these three pathways as the main targets in our study [22, 23]. Although CaMKII and PI3-K/AKT signaling activation were reported to be important, our present results showed that STDP treatment had little influence on the CAMKII and PI3K/AKT signaling pathways (Figures 5-7), while STDP treatment promoted the phosphorylation of ERK1/2 after ischemia. These results suggested that ERK1/2 mediated the STDP-induced cardioprotection against acute myocardial ischemia injury, in consistence with previous reports (Figure 6) [16]. Therefore, our findings contributed to understanding the molecular mechanisms of cardiac protection of STDP.

With more than 2 decades of clinical applications, clinical pharmacological studies have demonstrated STDP’s effects, including anti-AS, improving microenvironment, and protecting myocytes and vascular endothelial cells [24]. Moreover, long-term STDP intake was reported to significantly improve heart function and decrease angina pectoris attack as well as protect patients from myocardial infarction [25]. Our study showed that STDP had a dose-dependent inhibitive effect on the elevation of cTnI, CK, CK-MB, LDH, and α-HBD during myocardial ischemia.

Since there are 13 major active ingredients in STDP, one may possibly postulate that STDP could work on multiple mechanisms of myocardial ischemia simultaneously. It was reported that STDP could reduce pituitrin induced acute myocardial ischemia via an anti - apoptotic signaling pathway, down-regulating expression of Bax and up-regulating expression of Bcl - 2 in myocardial tissue [26]. Moreover, STDP was reported to attenuate atherosclerotic lesions in ApoE(-/-) mouse model via reducing the aortic expression of miR-21a, miR-132, miR-126a, miR-155 and increasing expression of miR-20a [16]. However, numerous studies showed that Compound Chinese Medicine could take one aspect effect through a single signaling pathway, such as Wenxin Keli, Salvionate, and Tongxinluo capsule [27–29]. In the present study, it was showed that STDP worked via a single signaling pathway, in consistence with these publications [27–29]. Thus, this study provided a mechanism for the clinical STDP treatment in improving cardiac function of angina pectoris patients.

To supplement molecular studies on the mechanisms we used pharmacological inhibitors. However, these inhibitors might have limited specificity and completeness in blocking their target proteins. Further studies combining pharmacological and genetic evidences may be needed to supports our hypothesis that ERK1/2 is a key downstream molecular mediator of STDP in the RISK signaling pathway.

In summary, the present study showed that STDP reduced the ISO-induced myocardial ischemic injury. This result experimentally proved the effectiveness of STDP as a clinical therapy to protect against ischemia injury at an early stage. These experimental evidences of the STDP-induced cardio-protection can help to explain the improved outcomes in patients with acute coronary syndrome treated with STDP and may lead to the development of better drugs and/or new therapeutic applications of STDP.

## MATERIALS AND METHODS

### Animals and reagents

This study was performed in accordance with the guidelines and with approval from the Institutional Animal Care and Use Committee of Guangdong Province Hospital of Chinese Medicine, Guangzhou University of Traditional Chinese Medicine, and with the Guide for the Care and Use of Laboratory Animals (Department of Health and Human Services, National Institutes of Health, Publication No. 86-23, revised 1996).

10-12 weeks male wild-type SD rats (200 ± 5 g body weight) were obtained from the Experimental Animal Center of Guangdong Province. STDP was provided by Inner Mongolia Conba Pharmaceutical Co., Ltd. (Inner Mongolia, China).

### Rat myocardial ischemic injury model *in vivo*

A model of myocardial ischemic injury was constructed by isoproterenol (ISO, 85 mg/kg/day for 2 days) subcutaneous administration (i.h) in rats. Serum levels of cardiac troponin I (cTnI), creatine kinase (CK), CK-MB, lactate dehydrogenase (LDH), aspartate transaminase (AST), α-hydroxybutyric dehydrogenase (α-HBD) were detected. Rats were assigned to four groups: CON (n=9), ISO (n=8), MET (n=6), and STDP (n=9). Rats in CON group received saline i.p and all the procedures except ISO administration; rats in ISO group were subjected to ISO administration (i.p) for 2 days; MET rats received metoprolol, 10 mg/kg/day i.p, for 3 days prior to and 2 days after ISO stimulation (totally 5 days), to serve as positive controls of the protective effects of ischemia treatments. Based on our previous study, literature, and clinical usage in patients (with dose conversion between humans oral usage and animals), STDP powder at a dose of 3 g/kg body weight mixed with 1 mL saline was administered daily via direct gastric gavage, for 3 days prior to and 2 days after ISO stimulation (totally 5 days).

### ELISA analysis

At the time of sacrifice (the day after ISO i.p for 2 days) and under anesthesia, 1 ml of blood was collected from the inferior vena cava of each rat and immediately centrifuged at 3000 revolutions per minute for 15 minutes. The plasma supernatant was recovered and kept at −80 °C until performing ELISA assays of cTnI, CK, CK-MB, AST, LDH, and α-HBD concentrations at 25 °C on a Hitachi 7180 analyzer. Following assays were performed according to manufacturer’s protocols: CK ELISA kit (Ref#12132672), CK-MB ELISA kit (Ref#12132893), cTnI high sensitive ELISA kit (Ref#05092728), AST ELISA kit (Ref#11876848), LDH ELISA kit (Ref#03002209), and α-HBD ELISA kit (Ref#11876937) (Roche Diagnostics, Indianapolis, IN, USA).

### Dose-dependent experiment of cTnI *in vivo*

To evaluate the dose-dependent effect of STDP on ISO-induced myocardial ischemia in rats, five concentrations of STDP (1, 10, 100, 1000 and 10000 mg·kg-1·day-1, respectively) were used in 5 separate animal groups: STDP-1 mg group (n=9), STDP-10 mg group (n=9), STDP-100 mg group (n=9), STDP-1000 mg group (n=9), and STDP-10000 mg group (n=6). cTnI concentrations was detected as a golden indicator of myocardial injury by using a cTnI high sensitive ELISA kit on a Hitachi 7180 analyzer. Dose-response curve of DLT was analyzed.

### Rat H9C2 cardiomyocyte culture *in vitro*

Rat H9C2 cardiomyocyte cell line was obtained from the American Type Culture Collection (ATCC, Manassas, VA, USA). H9C2 cells were seeded in 6-well plates, and maintained in DMEM supplemented with 10% fetal calf serum at 37°C in CO_2_ incubation. The medium was replaced every 2–3 days, and cells were sub-cultured or subjected to experimental procedures at 80–90% confluence. Subsequently, the H9C2 cells were synchronized in serum-free DMEM 24 h before treatment. To evaluate cardio-protective effects of STDP on rat H9C2 cells, four groups were studied: Control, ISO, MET, and STDP. ISO group was subjected to 1 μM ISO for 30 minutes while Control group was stimulated with 0.2 ul DMSO. MET group (10uM, served as positive drug treatment) and STDP group (0.65 mg/ml) were pretreated for 2 hours followed by ISO stimulation. After ISO pre-incubation for 30 minutes, all cell samples were lysed in 150 μl cell lysis buffer for Western blot experiments. All experiments were performed in triplicate.

### Western blot analysis

The cardiac tissue cells were rinsed and homogenized in RIPA lysis buffer containing protease inhibitor PMSF. The insoluble protein lysate was removed by centrifugation at 12,000 rpm for 5 min at 4°C. 50 μg of the protein lysate was resolved using 12% SDS–polyacrylamide gel electrophoresis. The gels were transferred to polyvinylidene difluoride (PVDF) membranes by semidry electrophoretic transfer at 200 mA for 60 min. The PVDF membranes were blocked one hour in 5% milk at room temperature and subjected to Western blot analysis. Following antibodies were used: anti-phospho-ERK1/2 (Thr202/Tyr204), anti-phospho-PKB (Ser473), anti-phospho-CaMKII, and anti-PKB (Cell Signaling Technology, Beverly, MA, USA), anti-ERK1/2, anti-GAPDH and anti-eIF-5 (Santa Cruz Technology, Delaware, CA, USA). The sheets were analyzed with antibodies according to the supplier’s protocol, and visualized with peroxidase and an enhanced-chemiluminescence system (ECL kit, Pierce Biotechnology, Inc.). Bands were visualized by use of a super western sensitivity chemiluminescence detection system (Pierce, IL). Autoradiographs were quantitated by densitometry (Science Imaging system, Bio-Rad, Hercules, CA), and the ratio was compared between the phosphorylated p-ERK1/2, p-AKT, p-CaMKII and total proteins ERK1/2, AKT, CaMKII, respectively.

### Statistical analysis

Data are reported as means ± S.E.M. Bonferroni’s post hoc method was used to assess the significance of differences using GraphPad Prism version 4.0. A P-value of <0.05 was considered statistically significant.

## SUPPLEMENTARY MATERIALS FIGURES



## Abbreviations

STDP: Shexiang Tongxin dropping pill
ISO: isoproterenol
i.h: subcutaneous injection
ERK1/2: extracellular regulated protein kinases
PI3-K: phosphatidylinositol-3-kinase
CamKII: Ca2+/calmodulin-dependent protein kinase II
ELISA: enzyme linked immunosorbent assay
cTnI: cardiac troponin I
CK: creatine kinase
CK-MB: serum creatine kinase MB
LDH: lactate dehydrogenase
α-HBD: alpha-hydroxybutyrate dehydrogenase
AKT: protein kinase B
HW: heart weight
BW: body weight
TL: tibial length
IHD: ischemic heart disease
PD: PD98059
LY: LY294002
KN: KN-93
