# Supplementary material for: Shexiang Tongxin dropping pill protects against isoproterenol-induced myocardial ischemia in vivo and in vitro
Source: Oncotarget. 2017 Nov 14;8(65):108958–69. doi: 10.18632/oncotarget.22440 (PMC5752495; doi:10.18632/oncotarget.22440)
Supplement: Supplementary file 1 [file oncotarget-08-108958-s001.pdf]

Shexiang Tongxin dropping pill protects against isoproterenol-induced myocardial ischemia *in vivo* and *in vitro*

SUPPLEMENTARY MATERIALS

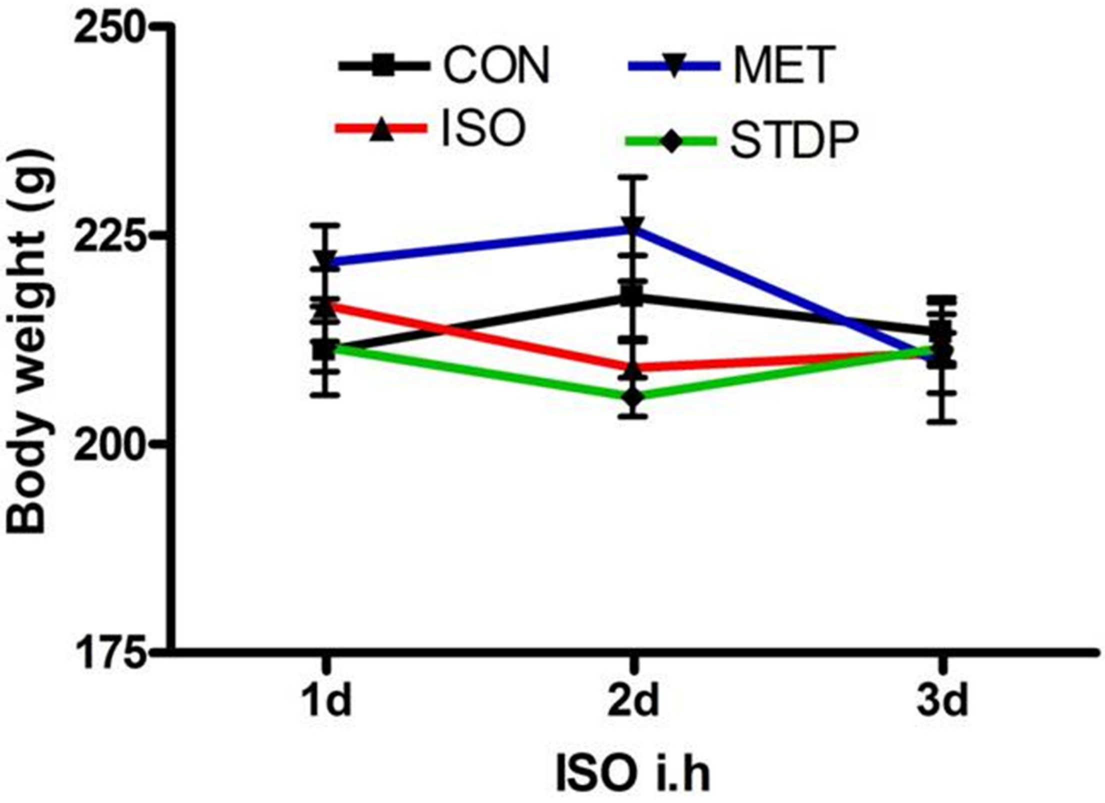

Supplementary Figure 1: BW compared among the four groups. Dynamic body weights were compared among CON, ISO, MET, and STDP group on 1st,2nd, and 3rd day after ISO administration i.h. There were no significant differences.

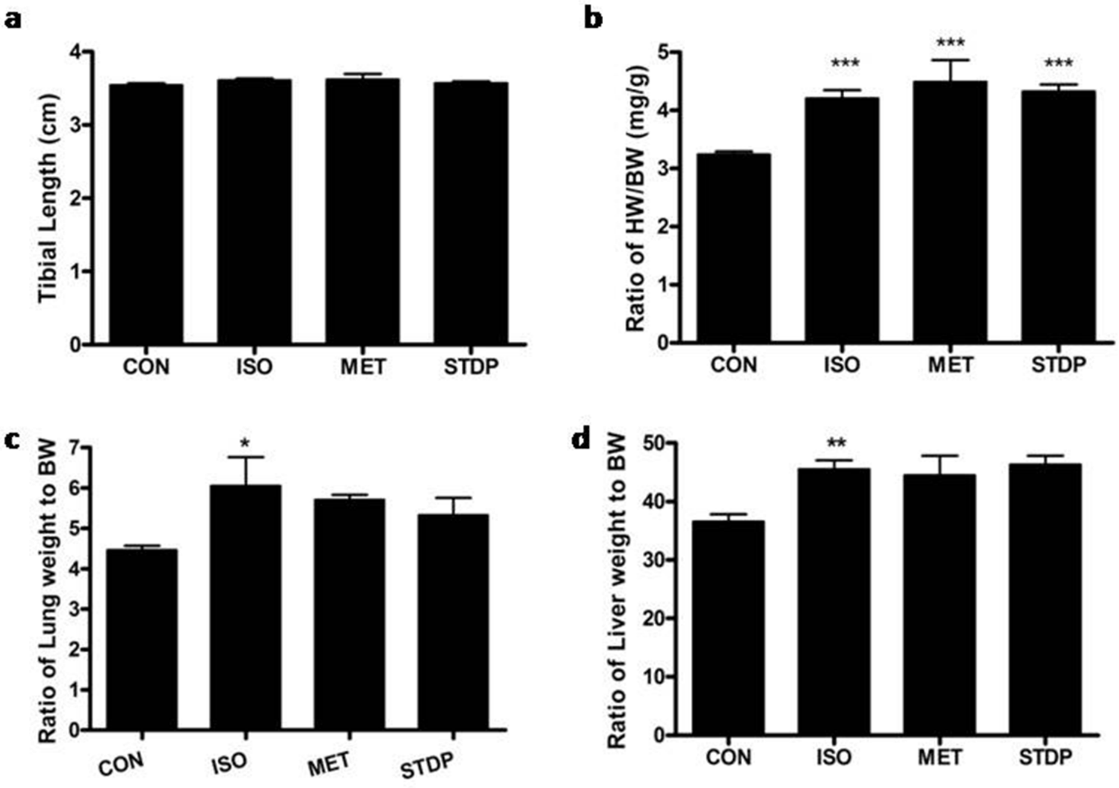

Supplementary Figure 2: Hearts, lungs, and livers were compared in the study. **(a)** Tibial length, **(b)** Ratio of HW/BW, **(c)** Ratio of lung weight/BW, **(d)** Ratio of liver weight/BW were detected from anatomic data. Data are presented as mean ± S.E.M. * $P < 0.05$, ** $P < 0.01$, *** $P < 0.001$ compared with CON group.

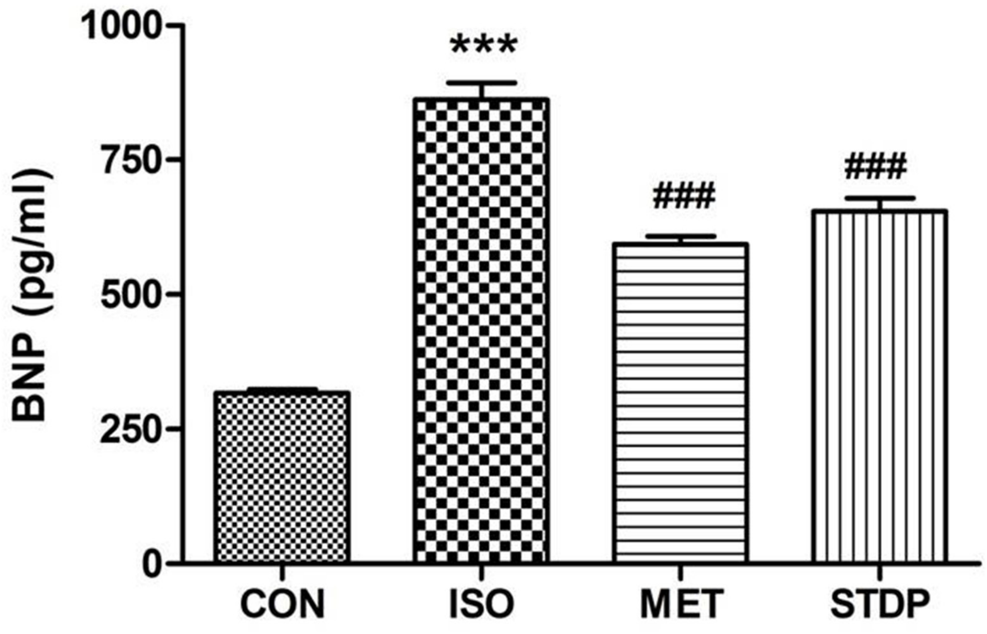

Supplementary Figure 3: The serum contents of BNP were measured *in vivo*. Plasma levels of BNP among the 4 study groups (CON, ISO, MET, STDP) were measured by ELISA methods. *** $P < 0.001$, compared with CON, ### $P < 0.001$, compared with ISO.
